# Systematic Review of Pooling Sputum as an Efficient Method for Xpert MTB/RIF Tuberculosis Testing during the COVID-19 Pandemic

**DOI:** 10.3201/eid2703.204090

**Published:** 2021-03

**Authors:** Luis E. Cuevas, Victor S. Santos, Shirley Verônica Melo Almeida Lima, Konstantina Kontogianni, John S. Bimba, Vibol Iem, Jose Dominguez, Emily Adams, Ana Cubas Atienzar, Thomas Edwards, S. Bertel Squire, Patricia J. Hall, Jacob Creswell

**Affiliations:** Liverpool School of Tropical Medicine, Liverpool, UK (L.E. Cuevas, K. Kontogianni, V. Iem, E. Adams, A.T. Atienzar, T. Edwards, S.B. Squire);; Federal University of Alagoas, Arapiraca, Brazil (V.S. Santos);; Federal University of Sergipe, Aracaju, Brazil (S.V.M. Almeida Lima);; Bingham University, Karu, Nigeria (J.S. Bimba);; National TB Control Program, Vientiane, Laos (V. Iem);; Institut d'Investigació Germans Trias i Pujol and Universitat Autònoma de Barcelona, Badalona, Spain (J. Dominguez);; Centers for Disease Control and Prevention, Atlanta, Georgia, USA (P.J. Hall);; Stop TB Partnership, Innovations and Grants, Geneva, Switzerland (J. Creswell)

**Keywords:** antimicrobial resistance, coronavirus disease, COVID-19, diagnostics, Xpert MTB/RIF testing, mycobacteria, pandemic, pooling, SARS-CoV-2, systematic reviews, tuberculosis, viruses, tuberculosis and other mycobacteria, severe acute respiratory syndrome coronavirus 2, bacteria, TB

## Abstract

GeneXpert-based testing with Xpert MTB/RIF or Ultra assays is essential for tuberculosis diagnosis. However, testing may be affected by cartridge and staff shortages. More efficient testing strategies could help, especially during the coronavirus disease pandemic. We searched the literature to systematically review whether GeneXpert-based testing of pooled sputum samples achieves sensitivity and specificity similar to testing individual samples; this method could potentially save time and preserve the limited supply of cartridges. From 6 publications, we found 2-sample pools using Xpert MTB/RIF had 87.5% and 96.0% sensitivity (average sensitivity 94%; 95% CI 89.0%–98.0%) (2 studies). Four-sample pools averaged 91% sensitivity with Xpert MTB/RIF (2 studies) and 98% with Ultra (2 studies); combining >4 samples resulted in lower sensitivity. Two studies reported that pooling achieved 99%–100% specificity and 27%–31% in cartridge savings. Our results show that pooling may improve efficiency of GeneXpert-based testing.

Xpert MTB/RIF (Cepheid, https://www.cepheid.com) is a cartridge-based nucleic amplification assay for use with Cepheid’s GeneXpert diagnostic instrument systems that detects both *Mycobacterium* *tuberculosis* complex (MTB) and resistance to rifampin (RIF). In 2010, the World Health Organization endorsed Xpert MTB/RIF for laboratory detection of tuberculosis (TB) (*1*), signaling a sea change for diagnosing TB. Xpert MTB/RIF increased sensitivity over microscopy and its ability to simultaneously detect rifampin resistance led to its rapid adoption in low- and middle-income countries. Within the first 5 years, 23 million cartridges were procured at the negotiated price of $9.98/each (P. Jacon, Cepheid, pers. comm., email, April 2020). In 2017, the Cepheid Xpert MTB/RIF Ultra assay (Ultra) was released for use on GeneXpert instruments and results determined to be comparable to those from the Xpert MTB/RIF assay, with an even lower limit for detection (*1*). 

Coronavirus disease (COVID-19) is severely disrupting health systems and is threatening progress made by national TB control programs. The new Xpert Xpress SARS-CoV-2 test is run on the same GeneXpert instruments as those for Xpert MTB/RIF and Ultra testing; it is being expedited for large-scale production and deployment. Consequently, TB-testing capacity, already limited by the availability of necessary staff, testing modules, and Xpert MTB/RIF and Ultra cartridges, may be further reduced by the increased demand for GeneXpert for COVID-19 testing (*3*). There is an urgent need to develop laboratory testing approaches to expand TB diagnostic and case-finding services in preparation for crises, such as the COVID-19 pandemic. 

GeneXpert-based testing for TB requires 1 cartridge per sputum sample. However, screening for other infectious diseases has used sample pooling methods, in which samples from several patients are pooled together for a single test to optimize processing. If a pooled-sample test is negative, all samples in the pool are considered negative; if the pooled-sample test is positive, all samples in the pool are retested individually to identify the samples that are positive. This method is routinely used in situations where the prevalence of disease is low (e.g., blood banks screening donated blood for hepatitis and syphilis) (*4**–**9*). The method can substantially reduce workload and cost and, for TB, could more efficiently process samples for diagnosis. We reviewed the literature to determine the accuracy of pooling for Xpert MTB/RIF and Ultra detection of pulmonary TB, with the aim of supporting TB programs as they continue to test for TB in the context of increased resource constraints during the COVID-19 pandemic. 

## Methods 

We conducted a systematic review following the Cochrane Collaboration’s Diagnosis Test Accuracy Working Group protocol (https://methods.cochrane.org). Our primary aim was to describe whether testing using GeneXpert for pulmonary TB on pooled samples would result in similar numbers of patients being confirmed with TB as testing samples individually. Secondarily, we aimed to describe the advantages and disadvantages reported, such as savings in cartridges used and time required to process samples. 

We searched PubMed, CINAHL, Global Health, and Web of Science for publications from January 2010–March 2020 with no regional or language restrictions. We used the terms “GeneXpert” OR “Xpert” OR “Ultra” AND “tuberculos*” AND “pool*” AND “diagnos*” with associated subject headings and search terms without filters (Appendix Table). S.V.M.A.L. and K.K. eliminated duplicates, screened titles and abstracts, and read full texts to determine eligibility. We also searched for article references manually and for abstracts published at the 2019 Union World Conference of Lung Health. Studies were included if they presented original data, if data were not duplicated in other publications, and if the articles were not reviews or opinions. We excluded studies that pooled several samples from the same patient to increase the yield and those that included samples other than sputum. Given the paucity of studies, we included both those that directly processed patient samples and those that used leftover samples to prepare a specimen repository for bench evaluation of the pooling method. We read selected studies in full for data extraction; L.E.C. and V.S.S. resolved disagreements by consensus. 

Data extracted included study identifiers (author, year, country, and setting), methods (study design, pooling methods, number of participants, pooling ratio, number of pools, and type of test), and whether the pooled positive and negative test results coincided with those obtained through individual testing. Data are presented as sensitivity and specificity values, considering the individual GeneXpert test as the reference. Sensitivity was defined as the proportion of pooled samples correctly identified as positive when the pool contained at least 1 sample with a positive individual GeneXpert test. Specificity was defined as the proportion of pooled samples correctly identified as negative when all samples in the pool were negative in individual GeneXpert tests. Data are presented with 95% confidence intervals and ranges. 

We assessed the quality of the studies based on a further reference standard, the use of TB culture by any method, whether pooled results were recorded blind to the individual results and whether participants had been recruited consecutively to represent the range of disease severity. The quality of studies and the risk of bias were assessed by 2 independent reviewers (authors) using the QUADAS-2 (Quality Assessment of Diagnostic Accuracy Studies) guidelines (https://www.bristol.ac.uk/media-library/sites/quadas/migrated/documents/quadas2.pdf). We used Cochrane Collaboration Rev-Man 5.3 software (https://training.cochrane.org/online-learning/core-software-cochrane-reviews/revman/revman-5-download) to generate the graphs on the risk of bias (Appendix Figures 1, 2). Because the studies were highly heterogeneous and most (4/6) did not present data on specificity, we were unable to perform a meta-analysis to estimate the pooled sensitivity and specificity or to explore the reasons for heterogeneity through meta-regression. Institutional review board approval was not required because all data sources and publications were in the public domain and in aggregate format. 

**Figure 1:**
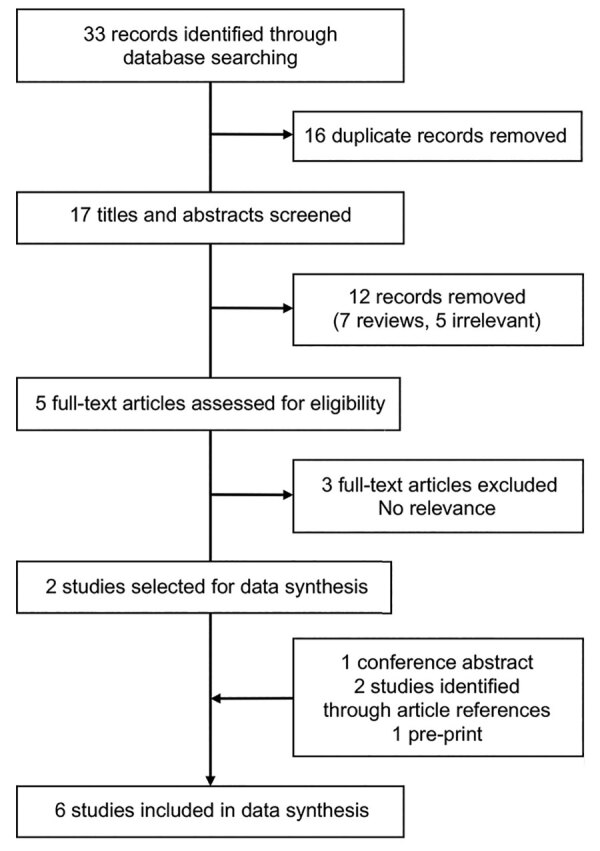
Flow diagram of study selection for a systematic review of pooling sputum as an efficient method for Xpert MTB/RIF and Ultra (Cepheid, https://www.cepheid.com) testing for tuberculosis during the coronavirus disease pandemic.

**Figure 2:**
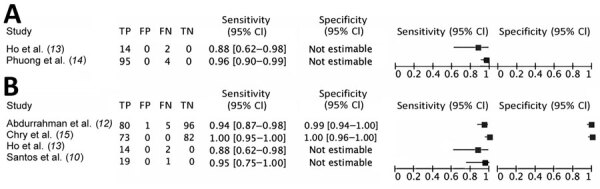
Sensitivity and specificity for pooling sputum in the ratio of 1:2 (A) and pooling sputum in the ratio of 1:4 (B) in a systematic review of pooling sputum as an efficient method for Xpert MTB/RIF and Ultra testing (Cepheid, https://www.cepheid.com) for tuberculosis during the coronavirus disease pandemic.

## Results 

We identified 33 publications through the initial publication search. After screening titles and abstracts, we assessed 5 full-text articles for eligibility and initially included 2 in data syntheses. In addition, 4 studies were identified from other sources: 1 conference report, 1 preprint article, and 2 articles from the reference lists of other studies. We included 6 articles in the final data synthesis (Figure 1). One study was conducted in South America (*10*), 2 in Africa (*11**,**12*), and 3 in Asia (*13*–*15*); all were published during 2014–2020, before the COVID-19 pandemic. 

We assessed the quality of the studies and the risk of bias (Appendix Figures 1, 2). Three studies used samples collected directly from patients with presumptive TB, and 3 studies used previously collected stored samples with known GeneXpert results. Studies pooling direct clinical samples were conducted in high-burden settings in which the proportion of patients that tested GeneXpert-positive was high (15%, 16%, and 38.6%), whereas stored samples were used to prepare pools varying the proportion of positive specimens in each pool to explore the effect on sensitivity. Pools were prepared with clinical samples from consecutive patients in 5 studies and in bench-prepared spiked sputum in a laboratory setting in 1 study. The latter study had also prepared the pool using combinations of smear-positive/culture-positive and smear-negative/culture-positive samples. Generally, the studies followed a similar approach to pooling: a sample was collected from patients with presumptive TB and split into aliquots for Xpert MTB/RIF or Ultra testing following the manufacturer’s guidelines. Studies that processed and homogenized sputum used the same steps for the individual and pooled GeneXpert tests. One aliquot was used to obtain an individual result, which was considered the reference result; and the second aliquot was mixed with aliquots from other patients and then tested as a pooled sample. All studies reported that laboratory technicians were blind to whether they were testing pooled versus individual samples. One study collected smear and culture results from all participants in addition to the GeneXpert result (*11*). Four studies tested sputum using Xpert MTB/RIF (*11*–*14*) and 2 with Ultra (*10*,*15*) (Table 1). 

**Table 1:** Characteristics of the studies, number of participants, and pool size used in a systematic review of pooling sputum as an efficient method for Xpert MTB/RIF and Ultra testing for tuberculosis during the coronavirus disease pandemic*

Study	Country	Participants recruited from	No. samples	Culture	GX cartridge used	Pooling ratio	No. pools	GX-pos,† no. (%)	GX-neg,† no. (%)	RIF- pos, no.	Comments
(*11*)	South Africa	Reference laboratory	100	Yes	MTB/RIF	1:5	20	20 (20.6)	80 (79.4)	5	Culture and SM pos
			85			1:5	17	17 (20)	68 (32)	3	Culture pos/SM neg
(*12*)	Nigeria	OPD	729	No	MTB/RIF	1:4	185‡	115 (15.8)	614 (84.2)	4	Compared active and passive case finding
(*13*)	Vietnam	SS	118	No	MTB/RIF	1:2	16	75 (63.6)	43 (36.4)	NR	None
						1:4	16				
						1:6	16				
						1:8	16				
						1:10	16				
						1:12	16				
(*14*)	Vietnam	Hospitals	262	No	MTB/RIF	1:2	101§	99 (37.7)	163 (62.3)	NR	Pools constructed 1 pos/1 neg
(*15*)	Cambodia	ACF	584	No	ULTRA	1:4	125	91 (15.6)	493 (84.4)	3	Used chest radiograph to screen
						1:3	28				
(*10*)	Brazil	Prisons, SS	1,120	Yes	ULTRA	1:4	20	100 (8.9)	1,020 (91.1)	NR	None
						1:8	20				
						1:12	20				
						1:16	40				

*Xpert MTB/RIF and Ultra, Cepheid (https://www.cepheid.com). ACF, active case finding; GX, GeneXpert; hosp, hospitalized patients; neg, negative; NR, not reported; OPD, outpatient department; pos, positive; RIF, rifampin; SM, smear; SS, spiked samples.

†Single tests.

‡3 had failed results.

§2 had failed results.

These 6 studies tested 1,878 individual samples. Participants were recruited from hospitals (n = 262), ambulatory clinics (n = 914), and outreach activities (n = 702). The percentage of individual patients with Xpert MTB/RIF-positive tests included in the pools ranged from 8.9% to 37%, except for 1 in vitro study, which used spiked samples and prepared pools with up to 64% of positive samples. Only 15 (0.8%) participants across all studies had rifampin resistance (Table 1). Overall, of the 690 pools tested, 117 pooled 2 samples, 28 pooled 3 samples, 364 pooled 4 samples, 37 pooled 5 samples, 16 pooled 6 samples, 36 pooled 8 samples, 16 pooled 10 samples, 36 pooled 12 samples, and 40 pooled 16 samples. Most of the pools with high numbers of samples (≥6) per pool were in the bench-based study. Only 2 studies reported specificity, 1 in which pools were tested with Xpert MTB/RIF (99%, 95% CI 94%–100%) and 1 in which pools were tested with Ultra (100%, 95% CI 96%–100%; Table 2) (*12*,*15*). 

**Table 2:** Tuberculosis Xpert results of pools composed of positive and negative samples, with sensitivity and specificity, in a systematic review of pooling sputum as an efficient method for Xpert MTB/RIF and Ultra testing for tuberculosis during the coronavirus disease pandemic

Study	Pooling ratio	Test results, no.				Sensitivity, % (95% CI)	Specificity, % (95% CI)
True pos†	False pos‡	False neg†	True neg‡
(*11*)	1:5 (Cult neg/SM pos)	20	NA	0	NA	100 (80–100)	NR
	1:5 (Cult pos/SM neg)	13	NA	4	NA	76 (50–92)	NR
(*12*)	1:4	80	1	5	96	94 (87–98)	99 (94–100)
(*13*)	1:2	14	NA	2	NA	88 (62–98)	NR
	1:4	14	NA	2	NA	88 (62–98)	NR
	1:6	11	NA	5	NA	69 (41–98)	NR
	1:8	10	NA	6	NA	63 (35–85)	NR
	1:10	13	NA	3	NA	81 (54–96)	NR
	1:12	13	NA	3	NA	81 (54–96)	NR
(*14*)	1:2	95	NA	4	NA	96 (90–99)	NR
(*15*)	1:4	73	0	0	80	100 (95–100)	100 (96–100)
(*10*)	1:4	19	NA	1	NA	95 (75–100)	NR
	1:8	20	NA	0	NA	100 (83–100)	NR
	1:12	16	NA	4	NA	80 (56–94)	NR
	1:16	39	0	1	0	98 (87–100)	NR

*Xpert MTB/RIF and Ultra, Cepheid (https://www.cepheid.com). Cult, culture, NA, not applicable; neg, negative; NR, not reported; pos, positive; SM, smear. †At least one of the patients included in the pool had an Xpert-positive test. ‡All patients included in the pool were Xpert-negative in the individual tests.

The 2 studies (*13**,**14*) combining 2 sputum samples per pool reported 87.5% and 96.0% Xpert MTB/RIF sensitivity relative to individual testing (Figure 2, panel A). The 4 studies combining 4 samples per pool reported sensitivities of 88% (*10*) and 96% (*12*) for Xpert MTB/RIF and 95% (*13*) and 100% (*15*) for Ultra (Figure 2, panel B). In 2 studies (*10*,*13*), pools combining >4 sputum samples reported lower sensitivity ranges for Xpert MTB/RIF (63%–81%) and for Ultra (80%–100%) (Table 2). 

Given that all studies had <200 pools, we combined the results from all studies with similar pool sizes and test type (e.g., all studies that pooled 4 samples and test them using Xpert MTB/RIF) to evaluate the effect of the number of pooled samples on accuracy. Although this approach has limitations due to variations in study design and proportion of sample positivity, we believe the benefit of this preliminary analysis of the potential use of pooling during the COVID-19 pandemic outweighs these limitations. After combination, when using Xpert MTB/RIF, 114/117 2-sputa pools and 101/201 4-sputa pools tested contained an Xpert MTB/RIF-positive sputum; when using Ultra, 93/173 4-sputa pools tested contained an Ultra-positive sputum. If only pools containing a positive sputum sample were considered, 109/114 2-sputa pools tested by Xpert MTB/RIF had a MTB-positive result (sensitivity 93.2%, 95% CI 87.0%–96.4%), and 94/101 4-sputa pools tested by Xpert MTB/RIF had a MTB-positive result (sensitivity 93.0%, 95% CI 86.4%–96.6%). Lastly, 92/93 of the 4-sputa pools tested by Ultra had an MTB-positive result (sensitivity 98.9%, 95% CI 94.1%–99.9%), an increase in sensitivity over those tested by Xpert MTB/RIF. 

Studies reported slight changes in the cycle threshold (C_t_) values of the pooled samples compared with the individual tests. Most of the C_t_ changes were relatively small, although studies were not sufficiently powered to determine statistical significance. One study reported that the pooled Xpert MTB/RIF test was negative in 5/10 samples with very low individual Xpert MTB/RIF semiquantitative results (*12*). The South African study that used reconstituted processed sputa to generate pools reported that 20 pools containing 1 smear-positive and 4 smear-negative, but culture-positive, samples yielded a median Xpert MTB/RIF C_t_ value increase of 12 (IQR 0.3–20.0), and 22 pools containing only smear-negative/culture-positive samples had a median C_t_ increase of 6.2 (IQR 3.2–16.0) (*11*). Another study (*13*) also reported that Xpert MTB/RIF C_t_ values increased slightly with increasing pool ratios and, although most pools had C_t_ values similar to the individual sample tests, pools containing >12 sputum samples had a median increase in C_t_ value of 2.1 (IQR 0.0–4.5). 

A study from South Africa (*11*) reported 5 five-sample pools in which 1 was smear-positive/culture-positive and RIF-resistant and 3 five-sample pools in which 1 was smear-negative/culture-positive and RIF-resistant. All 8 pools containing RIF-resistant samples tested positive for RIF-resistance (*11*). However, in Chry et al. (*15*), of the 3 MTB-positive/RIF-resistant samples subjected to Ultra testing, the pools containing the samples yielded MTB-positive but RIF-sensitive results. Abdurrahman et al. (*12*) included MTB-positive/RIF-resistant samples in all 4 pools, of which 3 were detected by Xpert MTB/RIF as MTB-positive/RIF-resistant and 1 as MTB-positive/RIF-sensitive. 

Only 2 studies (*12*–*15*) reported on the operational effects of using a pooling method, including cartridge costs and time savings. The 2 studies (*12**,**15*) using 4 samples per pool reported savings in cartridge costs alone of 31% ($2,295 on 230 Xpert MTB/RIF cartridges) and 27% ($2,092 on 202 Ultra cartridges). These 2 studies also reported reductions of 377 (62%) and 226 (26%) hours in the staff time required to process and run samples (Table 3). All 6 studies included comments indicating the pooling procedure was feasible and beneficial. The study from South Africa (*11*) noted the lower sensitivity found among smear-negative/culture-positive patients. Several studies mentioned the need for specific training on the pooling procedure. The only negative effect, reported anecdotally, was the need to process samples more carefully to avoid handling and reporting errors. No studies included data on patient outcomes, such as treatment initiation.

**Table 3:** Potential cost and time savings and positive and negative effects of pooling in a systematic review of pooling sputum as an efficient method for Xpert MTB/RIF and Ultra testing for tuberculosis during the coronavirus disease pandemic*

Study	Cartridge savings	Time savings, h (%)	Negative effects	Positive effects
(*11**)*	Model of 1,000 patients with TB prevalence rate of 3% found 67.5% cartridge savings	NR	Lower sensitivity for smear-negative tuberculosis; requires laboratory infrastructure and training	Processes higher volume of samples with fewer materials; time savings
(*12*)	11% cartridge savings for hospital-based patients	377 (62%)	Steps involved heighten potential for errors	High-level agreement with individual Xpert results at reduced cost; substantial time savings to process hospital samples
	41% cartridge savings for patients identified through active case finding	NR	NR	Higher savings on cartridge cost and processing time for patients identified through active case finding
(*13*)	NR	NR	NR	Improved feasibility and cost-effectiveness of large-scale testing; reduced number of cartridges
(*14*)	NR	NR	Increase in “error” results when using less buffer for pooling compared with standard buffer technique	Reduced costs and number of cartridges
(*15*)	27% (lower savings estimate using combination of approaches)	226/876 (26%) for all samples; 300/876 (30%) if hybrid approach used	NR	Method feasible; potential to reduce costs, increase throughput. Pooling can be used selectively if another screening test (e.g., radiograph) used for additional savings (hybrid approach)
	34.5% (if used in patients with normal chest x-rays)	NR	NR	Higher savings if only samples from patients without abnormal chest radiographs are included
(*10*)	NR	NR	NR	Method sensitive and cost-effective
*NR, not reported.				

## Discussion 

This systematic review synthesizes the available literature on the performance of the pooling method using sputum for GeneXpert testing for detecting pulmonary TB. Although the number of studies is small, the studies reported high sensitivity and specificity for 1:2 and 1:4 pooling ratios, replicating single test results, but pooling >4 samples decreased sensitivity. Studies reporting C_t_ values consistently reported a slight increase in C_t_ values and corresponding lower MTB/RIF semiquantitative results for pooled samples. This result is to be expected because testing samples together necessarily dilutes individual samples. Efficiency gained by pooling samples could increase the resilience of TB diagnostic services in a time when health system resources are being challenged by the COVID-19 pandemic. 

The Xpert MTB/RIF Ultra cartridge was expected to help improve the sensitivity of pooled tests because the new assay has a much lower limit for detection than Xpert MTB/RIF (*16*). Ultra’s improved performance was confirmed by the higher sensitivities reported in 2 studies included in this review, suggesting that Ultra may be preferred over Xpert MTB/RIF for pooled sample testing (*10*,*15*). Moreover, the only 2 studies reporting specificity (of 99% and 100%) indicated that almost all pools containing all negative individual samples correctly reported negative results for the pooled samples (*12*–*15*). This is an important consideration because the additional steps required to split sputum samples and the need to keep track of sputum batches with a link between individual samples could be prone to cross contamination and error. Further studies are needed to replicate these findings under operational conditions. 

Regarding the reproducibility of RIF resistance results in pooled samples, in 1 study from South Africa, all 8 individual RIF-resistant results were detected as pooled RIF-resistant (*11*). However, in a study in Cambodia, 3 samples with RIF-resistant results from individual testing were reported as RIF-susceptible in the pooled testing (*15*) and in a study from Nigeria, pooling missed 1 of 4 RIF-resistant results (*12*). Although pooling seems to be an unreliable method to detect RIF resistance, in practice all samples from MTB-positive pools would be retested individually, which should replicate RIF resistance results from individual samples. 

Almost all studies reported anecdotal positive feedback from laboratory staff, and 2 studies (*12**,**15*) quantified savings in cartridge costs and staff time required to process samples. Although both of those studies reported substantial savings, they were conducted in populations with a high proportion of patients testing positive. If a high proportion of presumptive TB patients is expected to be positive, presumably a greater proportion of pools would test positive and require follow-up testing of individual samples. Savings therefore would be more substantial when applied within outreach case-finding activities in the community, where typically around 5% of samples are Xpert MTB/RIF-positive (*12*) and lower in referral and congregate centers (e.g., prisons), where patients might have a higher probability of having TB. The expected proportion of positive samples may therefore guide the pooling ratio selected for evaluation. For example, in active case finding, it is likely a pool ratio of 1:4 would be highly efficient and generate substantial savings, whereas a ratio of 1:2 would be more suitable for busy TB diagnostic centers where the proportion of samples that are positive can be as high as 15%. Pooling is not likely to be useful at a much higher prevalence than 20%, because most of the pools would be positive and samples would have to be retested individually (B.G. Williams, unpub. data, https://arxiv.org/abs/1007.4903). Moreover, there are operational issues that need further study, as it is unclear whether the timing of sputum splitting could affect results. For example, splitting samples before adding the GeneXpert buffers requires dividing thick and infectious samples, which are likely to have unevenly distributed bacilli, whereas splitting after adding the buffers could increase the risk of cross contamination but provide a safer and more liquid sample with more evenly distributed bacilli. 

To inform national programs, further research is needed to determine the effects on time savings from pooled testing, from sample collection to notification and treatment initiation. Two studies quantified large reductions in testing time from pooling (*12**,**15*), which could shorten turnaround times for patient notification, but time to notification was not reported in any of the studies. Quality management of the pooling process is critical, as reflected in discussions in the studies highlighting the importance of sample management and procedure training. As with routine testing procedures, ensuring that pooling is implemented in a biosafe and quality-assured manner would help mitigate risk to laboratorians from increased sample manipulation and prevent errors in sample handling and testing, which could reduce efficiency and benefit to both patients and programs. 

Our findings are especially relevant during the ongoing global COVID-19 pandemic, which is severely disrupting health services, the availability of diagnostic and treatment resources, supply chains, and other disease control efforts. Although the diagnosis of COVID-19 takes precedence, steps can be taken to preserve key services for diagnosing and treating patients with presumptive TB. Quarantine and restriction of movement during the pandemic have limited accessibility to services and reduced the numbers of patients attending TB diagnostic and treatment centers. Confinement of the population to households and the resulting increase in contact with other household members in crowded conditions could increase TB transmission. A surge in undetected cases, together with increases in treatment interruptions, will likely lead to increases in incident cases. Demand for testing also may cause severe resource constraints. Preparing for this scenario, such as by introducing pooling strategies, may result in more efficient use of limited resources. 

Before the COVID-19 pandemic, the World Health Organization issued guidelines promoting a rapid diagnostic test, such as a GeneXpert-based test, for all persons with presumptive TB (*17*). However, <20% of the GeneXpert TB tests necessary to test the estimated 100 million people who develop presumptive TB each year have been procured (*2*). Individual rapid molecular diagnostic testing for all patients with presumptive TB remains the standard of care and a goal for national TB programs worldwide, but the cost of individually testing all estimated symptomatic persons using GeneXpert would have been more than US $1 billion in cartridges alone in 2018 (*2*), more than the total amount of funding provided by international donors globally for TB in 2019 (*18*). Moreover, although passive case finding has long been the standard approach in many countries, it is becoming apparent that outreach beyond health facilities is needed to identify those with TB missed by programs (*19*). Increasing outreach activities usually means more testing, requiring more cartridges, will be needed. However, a typically greater negative-to-positive testing ratio in persons identified through outreach activities means that pooling strategies might decrease costs. 

Despite the potential usefulness of our findings, the quality of evidence we present remains insufficient to support wide adoption of the pooling method. Because the 6 studies were heterogeneous, we were unable to conduct a meta-analysis, and we considered all the studies together with bench evaluations of the technical sensitivity and specificity of the methods; our findings should therefore be considered hypothesis-generating to promote and inform further studies. Moreover, all studies were underpowered for investigating the performance of the pooled testing method in subpopulations (e.g., HIV-positive vs. HIV-negative, men vs. women), and very few samples tested rifampin resistant. C_t_ values also need to be interpreted with caution. 

Although both Xpert MTB/RIF and Ultra tests report C_t_ values, the test algorithms that determine their C_t_ and semiquantitative results differ, which impacts the interpretation of C_t_-based analyses. Moreover, because C_t_ ranges vary between multiple tests on the same homogenized sample, it would have been preferable to describe changes in positivity relative to the semiquantitative results. However, semiquantitative results were not reported in most studies. Similarly, although culture was used in some of the studies, this information was not used to stratify analyses. A second reference method would have been useful to further investigate whether discordant results were potentially due to improper sample management, cross-contamination in the laboratory, or random variation due to the bacilli not being homogeneously distributed in the sputum sample. 

Despite these limitations, we propose that the pooling method be considered as an interim option to strengthen capacity of TB laboratories during times of crisis, such as during the COVID-19 pandemic. Our team is currently conducting accelerated evaluations of the pooling method in Laos and Nigeria. We encourage the TB community to conduct studies on the pooling strategy and other resource-saving strategies for TB diagnostic testing that generates data for open access databases to inform national programs. 

Appendix Additional information for systematic review of pooling sputum as an efficient method for Xpert MTB/RIF tuberculosis testing during the COVID-19 pandemic.
