# Supplementary material for: Systematic Review of Pooling Sputum as an Efficient Method for Xpert MTB/RIF Tuberculosis Testing during the COVID-19 Pandemic
Source: Emerg Infect Dis. 2021 Mar;27(3):719–27. doi: 10.3201/eid2703.204090 (PMC7920689; doi:10.3201/eid2703.204090)
Supplement: Appendix — Additional information for systematic review of pooling sputum as an efficient method for Xpert MTB/RIF tuberculosis testing during the COVID-19 pandemic. [file 20-4090-Techapp-s1.pdf]

Article DOI: https://doi.org/10.3201/eid2703.204090

Systematic Review of Pooling Sputum as an Efficient Method for Xpert MTB/RIF Tuberculosis Testing during the COVID-19 Pandemic

Appendix

Appendix Table. Full search strategy for literature to be included in systematic review

Database	Query	Items found
PubMed/Medline	“GeneXpert”[All Fields] OR “Ultra”[All Fields] AND “tuberculos*”[All Fields] AND “pool*”[All Fields] AND “diagnos*”[All Fields]	8
Web of Science	((GeneXpert OR Ultra) AND Tuberculos* AND Pool* AND Diagnos*)	16
Global Health	GeneXpert AND Tuberculos* AND Pool* AND Diagnos*	5
Cinahl	GeneXpert AND Tuberculos* AND Pool* AND Diagnos*	4

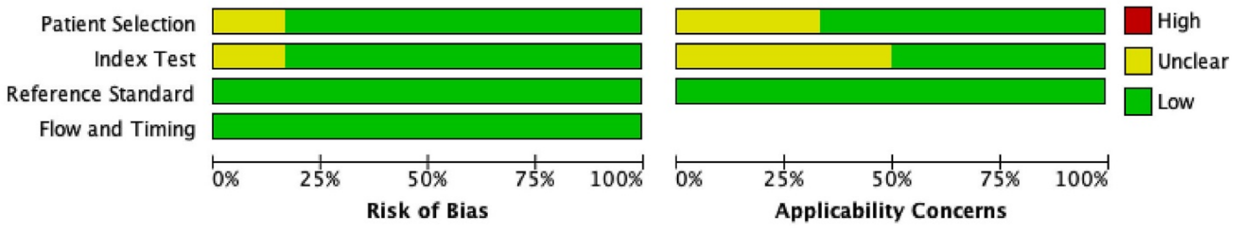

Appendix Figure 1. Risk-of-bias and applicability concerns graphs of included studies on Pooling Xpert test. Each methodological quality item is presented as percentages across all included studies. Figure was generated using Review Manager Version 5.3; Cochrane Collaboration.

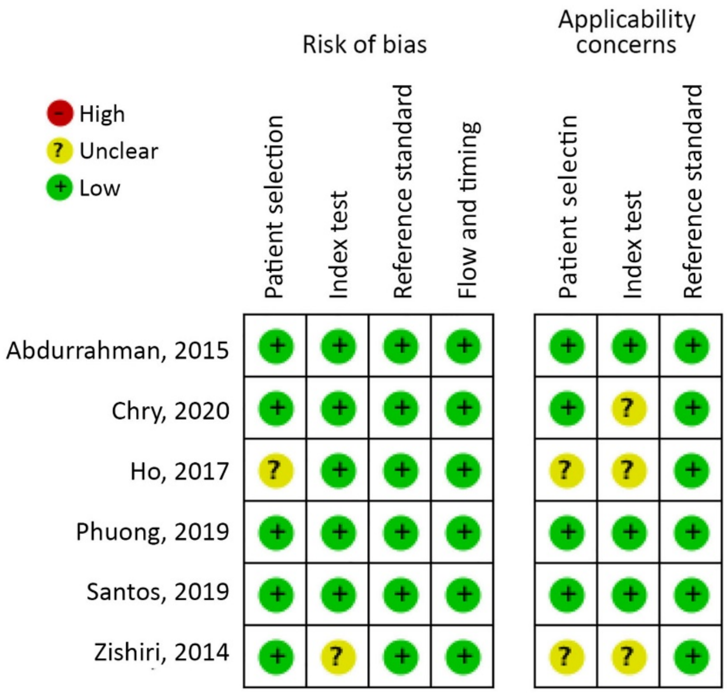

Appendix Figure 2. Summary for risk of bias of included studies on Pooling Xpert test. The green symbols represent low risk of bias, the yellow symbols represent unclear risk of bias, and the red symbols represent high risk of bias. The figure was generated using Review Manager Version 5.3; Cochrane Collaboration.
